# Effect of professional certification on employees’ return-to-work rate after occupational injuries in Korea: focusing on vulnerable groups

**DOI:** 10.1186/s12199-020-00930-0

**Published:** 2021-01-12

**Authors:** Suk Won Bae

**Affiliations:** 1 Department of Public Health, Graduate School, Yonsei University, 50 Yonsei-ro, Seodaemun-gu, Seoul, 03722 Republic of Korea; 2 The Institute for Occupational Health, Yonsei University College of Medicine, Seoul, 03722 Republic of Korea

**Keywords:** Occupational injury, Workers’ compensation insurance, Return to work, Certification

## Abstract

**Background:**

One effective way to improve return-to-work (RTW) performance may be to convince the employer that the worker has the necessary skills. The aim of this paper is to investigate the effect of having a professional certification among workers injured in occupational injuries on their return to work.

**Methods:**

The Panel Study of Workers’ Compensation Insurance (PSWCI) targets workers who completed medical care in 2012 after an occupational injury. The study population (*n* = 2000) was stratified by gender, age, region, disability grade, and rehabilitation service use. A total of 1458 workers were finally selected for this study.

The effect of having a certification on RTW status was calculated with an odds ratio and 95% confidence intervals using binomial and multinomial logistic regression analyses. In the binomial logistic regression analysis, the RTW group was made up as a combination of the return to original work and the reemployment groups.

**Results:**

The ORs of RTW among those with a certification compared to those without certification were 1.38 (1.16–1.65) in Model 1, 1.25 (1.05–1.50) in Model 2, and 1.22 (1.01–1.47) in Model 3. Among female workers with a certification, the OR of RTW was 4.60 (2.68–7.91), that of return to original work was 3.21 (1.74–5.91), and that of reemployment was 5.85 (3.34–10.27). Among daily workers with a certification, the OR of RTW was 1.32 (1.03–1.69) and that of reemployment was 1.37 (1.07–1.76).

**Conclusion:**

In conclusion, injured workers with a certification generally had a higher RTW rate. In particular, the RTW rate was higher among female workers and daily workers with a certification than among those without.

## Introduction

The Korean Ministry of Employment and Labor reported in 2018 that the number of occupational injured workers who consequently required the minimum 4 days of leave was approximately 100,000 [1]. This figure represents about 0.54% of 19 million workers who were eligible for Workers’ Compensation Insurance [1]. Even though this figure increased from 2017 to 2018, the overall trend of occupational injured workers has decreased [2]. However, regardless of such slight rises and falls, the total number of injured workers remains at a high level [1, 2]. In addition, the economic loss due to occupational injuries is significant [3, 4]. The direct compensation provided for occupational injuries was estimated at KRW 5 trillion (4.4 billion USD), and the estimated total economic loss, including indirect compensation, was KRW 25 trillion (21.8 billion USD) [1, 3]. Furthermore, workforce loss due to occupational injuries and economic losses are closely related [4–7].

Korea has implemented the Industrial Accident Compensation Insurance Act to provide prompt and fair compensation and support for rehabilitation and social return of disaster workers in the wake of a disaster [4]. The Korean Workers’ Compensation and Welfare Service (KCOMWEL) provides disability benefits to injured workers to compensate for the loss of income due to an accident [8, 9]. For individuals who are injured and not working, receiving immediate financial support and rehabilitation is critical. However, these forms of primary support are not enough to ensure employees maintain their previous work performance levels after the accident. The accident causes injured workers not only to lose the ability to work but also to experience loss of self-esteem and self-efficacy and depression, which hinders their return-to-work (RTW) rate [4, 10–15].

Several studies have identified factors affecting the RTW rate of injured workers [16–19]. These factors can be divided into five categories: personal, socioeconomic, occupational, degree of injury, and employer interest level in workers’ reemployment [4, 16, 19]. RTW is known to be associated with personal factors such as being male, being younger, being married, having a higher education level, and having a higher household income [18] and occupational factors such as larger-sized workplaces, longer duration of employment, regular workers, and other factors such as lower disability ratings after occupational injuries, shorter length of hospital stays and recovery duration, hospitals with higher quality scores, and greater interest of doctors in charge and employers in injured workers’ RTW [20, 21]. However, the above factors are outside the individual’s control and are hard to change even if the workers have strong will to RTW. Thus, it is necessary to study the factors within injured workers’ control that can positively affect RTW to assist in their RTW preparation.

One effective way to improve RTW performance is to convince the employer that the worker has the necessary skills. Professional certification in the field is often used as an official permit for performing a job [22, 23], and it can provide employers an objective evaluation of employees’ skills [22, 24]. Therefore, it has been recognized as a useful resource to help someone get a job [25–27]. It is possible that as an indicator of skills, professional certification could assist injured workers who want to RTW.

Most studies on occupational injuries focused on RTW [9, 18, 19]. However, few have examined the relationship between workers’ RTW and professional certification. Therefore, this study investigates the effect of professional certification on injured workers’ RTW after occupational injuries.

## Materials and methods

### Study design and participants

In this study, we used data from the first cohort (first–fifth waves) survey of the Panel Study of Workers’ Compensation Insurance (PSWCI) conducted by the Korea Workers’ Compensation and Welfare Service (KCOMWEL). The PSWCI provides useful data on all policy processes related to Industrial Accident Compensation Insurance Act, including those involving injured workers returning to work. The PSWCI targets workers who completed medical care in 2012 after an occupational injury (*n* = 82,493); the study population (*n* = 2000) was stratified by gender, age, region, disability grade, and rehabilitation service use.

Since the first survey in 2013, the PSWCI has been conducted annually. The fifth wave was completed in 2017. The study methods comprised one-to-one interviews conducted by professional interviewers who visited the participants; participants were asked to respond to the questionnaire in person [4, 28–32].

**Fig. 1:**
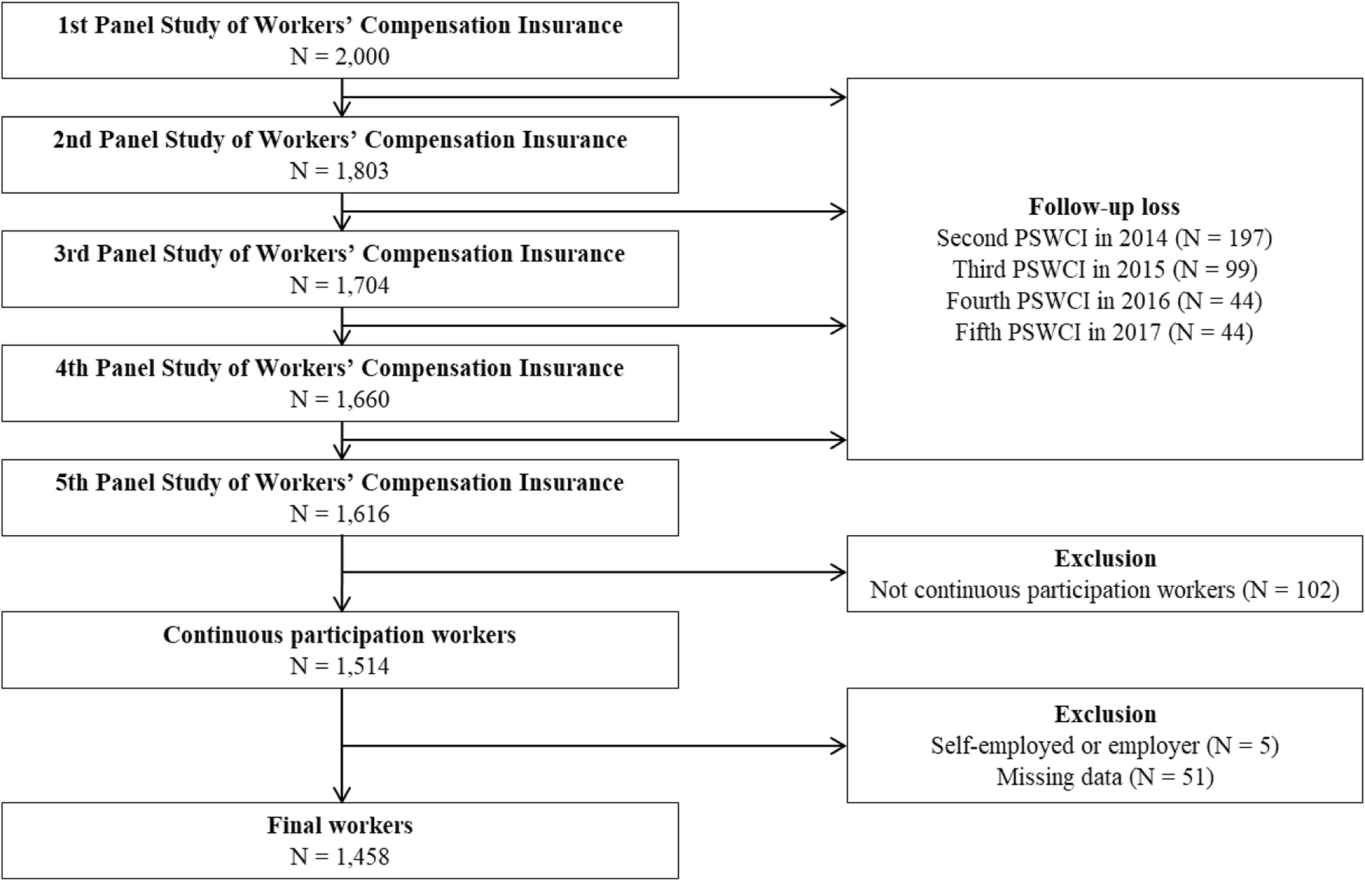
Schematic diagram of the study participants

Of the total 2000 participants in the first cohort survey, 1616 participated. Participants who had participated in the first–fifth waves were included in the present study. One hundred and two workers who had not continuously participated in the first–fifth waves were excluded. In addition, five workers who were self-employed or employers at the time of the occupational injury and 51 injured workers whose explanatory variables were not observed were also excluded. Therefore, a total of 1458 workers were finally selected for this study (Fig. 1).

### Sociodemographic characteristics

Age was classified into five groups: < 30, 30–39, 40–49, 50–59, and ≥ 60 years. Marital status was classified into three groups: “not married,” “married,” and “other” (separated, divorced, widowed). Education level was classified into three groups: “below high school graduate,” “high school graduate,” and “college graduate or above.”

### Occupational-related characteristics

Occupational factors included industry sector, status of workers, number of employees, and duration of employment in the workplace at the time of occupational injury.

Industry sector was categorized according to the Korean Standard Industry Classification (KSIC) (based on the International Standard Industry Classification, ISIC) into “manufacturing” and “construction”—which accounted for more than half of occupational injuries and diseases (manufacturing: 26.8%, construction: 27.1% in 2018 [1])—and “services (Information and communications; professional, scientific and technical activities, etc.)” and “other (Agriculture, forestry and fishing; Mining and quarrying, etc.).”

For worker status, full-time workers were classified as “regular workers,” whereas temporary and daily workers were classified as “daily workers.” The “number of employees in the workplace” was divided into four groups: < 5, 5–9, 10–29, and ≥ 30. The “duration of employment at the workplace” was divided into three groups: < 1 year, 1–3 years, and ≥ 3 years.

### Injury-related characteristics

Data on occupational injury type and disability rating were obtained from the KCOMWEL’s administrative database. The type of occupational injury was classified into injuries and diseases. If workers suffered any mental or physical disability from occupational-related injuries or diseases and met the requirements for industrial accident compensation, they were assigned a disability rating from Grades 1 to 14 under the Korean Industrial Accident Compensation Insurance Act. A grade close to Grade 1 indicates a more serious disability [4, 28]. Occupational injuries were classified into three categories: 1–7 (severe), 8–14 (moderate), and “none.”

### Qualifications-related characteristics

We investigated whether participants held any national certification (professional engineer, engineer, master craftsman, industrial engineer, craftsman, or other national certification), private degrees, or international (foreign) certificates (excluding general driving license). In the first wave of the panel study, participants with one or more certifications were coded as “yes,” and those with no certification were coded as “no.”

### Main outcome variables

The type of economic activity included return to original work, reemployment, self-employment, unpaid family work, unemployed, or economically inactive. Those who returned to their original work were workers who were injured at the workplace, but resumed work in the same position they held at the time of injury. Workers who found paid employment in a workplace other than where they experienced the injury were referred to as reemployed workers. Self-employed workers included private business owners or freelancers, and unpaid family workers referred to those who helped their family or relatives in the workplace for an average of > 18 h per week (more than 3–4 h per day). Unemployed persons referred to those who reported searching for a job more than once during the past 4 weeks and were able to work if they found a suitable job during the last 1 week; those who did not were classified as an “economically inactive population [20].”

In this study, the type of economic activity was classified as “return to original work,” reemployed and self-employed persons as “reemployed,” and unpaid family workers, the unemployed, and the economically inactive population as “non-return-to-work” (non-RTW).

### Statistical analyses

The characteristics of the participants regarding work status, and of those who returned to work with a certification, were analyzed using a chi-square test. The effect of having a certification on RTW status was calculated with an odds ratio (ORs) and 95% confidence intervals using binomial and multinomial logistic regression analyses. In the binomial logistic regression analysis, the RTW group was combined with the “return to original work” group and the “reemployed” group. In the multinomial logistic regression analysis, the non-RTW group was compared with the “return to original work” group and the “reemployed” group. All analyses were performed using the software SAS statistical package version 9.4 (SAS Institute, Cary, NC, USA).

## Results

The general participant characteristics according to RTW status are shown in Table 1. The return-to-original-work rate after occupational injuries was 38.1%, the reemployment rate was 51.2%, and the unemployment rate was 10.6% (*P* < 0.0001). The reemployment rates among male and female workers (51.6% and 49.4%, respectively) were higher than those for other RTW status (*P* = 0.0002). Regarding worker status, regular workers were found to have a higher rate of return to original work, whereas daily workers were found to have a higher reemployment rate (*P* < 0.0001). Those with a certification had a 42.7% return-to-original-work rate and a 50.3% reemployment rate (*P* = 0.0001).

**Table 1:** General characteristics of participants by return-to-work rate

	Returned to original work		Reemployed		Non-RTW		Total	*P* value *
	*N*	%	*N*	%	*N*	%		
Total	556	38.1	747	51.2	155	10.6	1458	< 0.0001
Age								< 0.0001
< 30	29	35.4	44	53.7	9	11.0	82	
30–39	103	49.5	95	45.7	10	4.8	208	
40–49	170	46.8	177	48.8	16	4.4	363	
50–59	191	36.5	279	53.2	54	10.3	524	
≥ 60	63	22.4	152	54.1	66	23.5	281	
Sex								0.0002
Male	472	39.2	621	51.6	110	9.1	1203	
Female	84	32.9	126	49.4	45	17.7	255	
Marital status								0.0001
Not married	73	35.8	112	54.9	19	9.3	204	
Married	436	41.3	510	48.3	110	10.4	1056	
Other	47	23.7	125	63.1	26	13.1	198	
Education level								< 0.0001
Less than high school	161	27.1	340	57.1	94	15.8	595	
High school	284	44.6	306	48.0	47	7.4	637	
College or above	111	49.1	101	44.7	14	6.2	226	
Industry								< 0.0001
Manufacturing	267	48.1	243	43.8	45	8.1	555	
Construction	59	15.3	274	71.0	53	13.7	386	
Service	88	47.6	80	43.2	17	9.2	185	
Other	142	42.8	150	45.2	40	12.1	332	
Status of workers								< 0.0001
Regular worker	449	55.3	311	38.3	52	6.4	812	
Daily worker	107	16.6	436	67.5	103	15.9	646	
Occupational injury type								0.0088
Injury	493	37.0	698	52.3	143	10.7	1334	
Disease	63	50.8	49	39.5	12	9.7	124	
Number of employees								< 0.0001
< 5	106	31.6	184	54.9	45	13.4	335	
5–9	100	29.1	211	61.3	33	9.6	344	
10–29	136	34.5	218	55.3	40	10.2	394	
≥ 30	214	55.6	134	34.8	37	9.6	385	
Duration of employment								< 0.0001
< 1 year	217	23.3	595	64.0	118	12.7	930	
1–less than 3 years	88	46.3	85	44.7	17	9.0	190	
≥ 3 years	251	74.3	67	19.8	20	5.9	338	
Disability rating								< 0.0001
1–7	18	29.5	22	36.1	21	34.4	61	
8–14	430	37.8	594	52.2	114	10.0	1138	
None	108	41.7	131	50.6	20	7.7	259	
Certification								0.0001
Yes	255	42.7	300	50.3	42	7.0	597	
No	301	35.0	447	51.9	113	13.1	861	

*Analyses using a chi-square test

The general characteristics of RTW workers were based on whether they held a certification (Table 2). The RTW rate among those with a certification was 93.0%, whereas the RTW rate among those without a certification was 86.9% (*P* < 0.0001). Overall, the RTW rate was higher among those with a certification except for those aged less than 30, with a marital status of “other,” educational level of “College graduate or above,” occupational injury type of “Disease,” duration of employment of 1–3 years, and disability rating of Grades 1–7.

**Table 2:** General characteristics of workers’ return-to-work rate by certification

	Yes		No		Total	*P* value *
	*N*	%	*N*	%		
Total	555	93.0	748	86.9	1303	< 0.0001
Age						< 0.0001
< 30	35	83.3	38	95.0	73	
30–39	113	94.2	85	96.6	198	
40–49	184	96.8	163	94.2	347	
50–59	173	92.0	297	88.4	470	
≥ 60	50	87.7	165	73.7	215	
Sex						< 0.0001
Male	501	93.1	592	89.0	1093	
Female	54	91.5	156	79.6	210	
Marital status						< 0.0001
Not married	96	88.9	89	82.8	185	
Married	414	95.0	532	90.7	946	
Other	45	84.9	127	95.1	172	
Education level						< 0.0001
Less than high school	122	89.1	379	82.8	501	
High school	299	94.6	291	90.7	590	
College or above	134	93.1	78	95.1	212	
Industry						0.0101
Manufacturing	212	95.1	298	89.8	510	
Construction	139	91.5	194	82.9	333	
Service	91	94.8	77	86.5	168	
Other	113	89.7	179	86.9	292	
Status of workers						0.0019
Regular worker	351	95.1	409	92.3	760	
Daily worker	204	89.5	339	81.1	543	
Occupational injury type						0.2083
Injury	501	93.3	690	86.6	1191	
Disease	54	90.0	58	90.6	112	
Number of employees						0.0043
< 5	106	93.0	184	83.3	290	
5–9	118	93.7	193	88.5	311	
10–29	169	93.4	185	86.9	354	
≥ 30	162	92.1	186	89.0	348	
Duration of employment						0.0038
< 1 year	324	92.1	488	84.4	812	
1–less than 3 years	70	89.7	103	92.0	173	
≥ 3 years	161	96.4	157	91.8	318	
Disability rating						0.0662
1–7	10	52.6	30	71.4	40	
8–14	445	94.5	579	86.8	1024	
None	100	93.5	139	91.5	239	

*Analyses using a chi-square test

Tables 3 and 4 show the ORs of RTW according to whether the participant had a certification. Model 1 was adjusted for gender and age. In Model 2, adjustments were made for the variables in Model 1 plus marital status, educational level, type of industry, worker status, number of employees, and duration of employment. Model 3 was adjusted for the variables in Model 2 plus disability rating and type of occupational injury. The ORs of RTW among those with a certification compared to those without certification were 1.38 (1.16–1.65) in Model 1, 1.25 (1.05–1.50) in Model 2, and 1.22 (1.01–1.47) in Model 3 (Table 3).

**Table 3:** Odds ratios of return-to-work rate by certification

	Return-to-work*											
	Model 1				Model 2				Model 3			
	OR	95% CI			OR	95% CI			OR	95% CI		
Certification												
Yes	1.38	1.16	−	1.65	1.25	1.05	−	1.50	1.22	1.01	−	1.47
No	1.00				1.00				1.00			

*Returned to original work group and reemployed group were integrated into RTW group

Model 1: Adjusted for age and sex

Model 2: Adjusted for Model 1 + marital status, education level, industry, status of workers, number of employees, and duration of employment

Model 3: Adjusted for Model 2 + disability rating and occupational injury type

**Table 4:** Odds ratios of return-to-work rate (return to original work, reemployed) by certification

	Returned to original work *												Reemployed *											
	Model 1				Model 2				Model 3				Model 1				Model 2				Model 3			
	OR	95% CI			OR	95% CI			OR	95% CI			OR	95% CI			OR	95% CI			OR	95% CI		
Certification																								
Yes	1.47	1.22	−	1.77	1.15	0.94	−	1.40	1.12	0.91	−	1.37	1.33	1.11	−	1.59	1.30	1.08	−	1.57	1.26	1.04	−	1.52
No	1.00				1.00				1.00				1.00				1.00				1.00			

*Compared non-RTW group with returned to original work group and reemployed group separately

Model 1: Adjusted for age, sex

Model 2: Adjusted for Model 1 + marital status, education level, industry, status of workers, number of employees, duration of employment

Model 3: Adjusted for Model 2 + disability rating, occupational injury type

The OR of return to original work among those with a certification was 1.47 (1.22–1.77) in Model 1, while no significant result was found in Models 2 and 3. The ORs of reemployment among those with a certification were 1.33 (1.11–1.59) in Model 1, 1.30 (1.08–1.57) in Model 2, and 1.26 (1.04–1.52) in Model 3 (Table 4).

Table 5 shows the ORs of RTW based on having a certification, which was stratified by age and status of workers. Among female workers with a certification, the OR of RTW was 4.60 (2.68–7.91), that of return to original work was 3.21 (1.74–5.91), and that of reemployment was 5.85 (3.34–10.27). Among daily workers with a certification, the OR of RTW was 1.32 (1.03–1.69) and that of reemployment was 1.37 (1.07–1.76).

**Table 5:** Odds ratio of return-to-work rate by certification (stratified analysis)

		Binomial *				Multinomial †							
		Return-to-work				Returned to original work				Reemployed			
		OR ‡	95% CI			OR ‡	95% CI			OR ‡	95% CI		
Sex	Male												
	Certification												
	Yes	0.92	0.74	−	1.13	0.89	0.71	−	1.13	0.93	0.75	−	1.16
	No	1.00				1.00				1.00			
	Female												
	Certification												
	Yes	4.60	2.68	−	7.91	3.21	1.74	−	5.91	5.85	3.34	−	10.27
	No	1.00				1.00				1.00			
Status of workers	Regular worker												
	Certification												
	Yes	1.05	0.78	−	1.42	0.97	0.71	−	1.32	1.09	0.79	−	1.49
	No	1.00				1.00				1.00			
	Daily worker												
	Certification												
	Yes	1.32	1.03	−	1.69	1.13	0.84	−	1.54	1.37	1.07	−	1.76
	No	1.00				1.00				1.00			

*Return to original work group and reemployed group were integrated into the RTW group

†The non-RTW group was separate compared with the return to original work group and reemployed group

‡Statistical data estimated from a binomial/multinomial multivariate logistic regression analyses that adjusted for all other covariates excluding an interesting variant

## Discussion

This study investigated the effects of holding a certification on RTW among occupationally injured workers after completion of medical care.

The ORs of RTW (4.60, 2.68–7.91), return to original work (3.21, 1.74–5.91), and reemployment (5.85, 3.34–10.27) among female workers with a certification were higher than in those with no certification. In addition, the ORs of RTW (1.32, 1.03–1.69) and reemployment (1.37, 1.07–1.76) among daily workers with a certification were higher than those with no certification (Table 5). Thus, the effect of having a certification on RTW is greater among female workers and daily workers who were relatively more vulnerable to a lower RTW rate than male workers and full-time workers, who are known to have a high RTW rate. This finding is consistent to the results of a previous study, which reported that having a certification had a positive effect (Coef. 1.468) on employment among vulnerable groups [27]. We therefore believe that having a certification is crucial for RTW in injured workers, especially those belonging to vulnerable groups.

Among sociodemographic factors, a higher educational level was found to be associated with a higher return-to-original-work rate. In terms of occupational factors, the return-to-original-work rate was higher among those who were in manufacturing industries, those who are regular workers, those whose workplace had more than 30 employees at the time of the occupational injury, and those who had worked for more than 3 years at the workplace where the occupational injury occurred. In terms of injury-related factors, non-disability or a lower disability grade was associated with a higher return-to-original-work rate (Table 1). This is consistent with previous findings in that the rate of return to original work was higher among students with high school graduation or more, manufacturing workers, regular and full-time workers, workers of workplaces with at least 50 employees at the time of occupational injury, workers whose duration of employment at the workplace at the time of occupational injury was 1 year or longer, and workers with no disability or with grade 10–14 disability [33–37]. In particular, the return-to-original-work rate among construction workers after an occupational injury was lower than among manufacturing and service workers. This was likely due to a higher percentage of daily workers in the construction industry than in the manufacturing and service industries [4, 38]. In addition, most construction workers were older and thus more likely to have a disability after a workplace accident [4].

With regard to the association between RTW and holding a certification, the RTW rate among workers with a certification was higher than among those without a certification (Table 2). The ORs of RTW among those with a certification were higher in Model 1 (1.38, 1.16–1.65), Model 2 (1.25, 1.05–1.50), and Model 3 (1.22, 1.01–1.47), indicating that the ORs of RTW in all models were higher among those with a certification, compared to those without one (Table 3). In addition, the OR of return to original work was 1.47 (1.22–1.77) in Model 1 (Table 4). This is consistent with the finding that RTW rate was higher among workers with a certification than among those without a certification [22, 24–26]. Therefore, it is clear that having a certification can affect RTW among injured workers. Although the KCOMWEL provides injured workers with customized rehabilitation support services across stages of medical care to facilitate their RTW and help the community at large [1–3], no support is offered for acquiring certification. We recommend that, as with vocational training support and employment support for injured workers, additional certification support should also be provided.

### Limitation and strength of the study

This study has several strengths. First, it is significant in that the results provide comprehensive data on various situations and needs of injured workers because they deal extensively with RTW and socioeconomic characteristics of injured workers. In addition, data from the only panel study in South Korea that includes occupationally injured workers were used. Second, the study examined the relationship between having a professional qualification and RTW and, for the first time, attempted to investigate the relationship among injured Korean workers.

The limitations of the study were as follows. In the PSWCI, eight types of certifications (national, private, and international) were surveyed. However, as the number of persons with each type of certification was low, separate analysis regarding the effects of each type was not feasible. Therefore, analysis was performed based only on whether someone had a certification. However, RTW among injured workers may have been affected by certification type. The data used in this study were classified according to the “Yes” or “No” of a certificate in the first wave of the PSWCI. The PSWCI did not investigate the year of obtaining a certificate, so they may have obtained a certificate after an occupational injury. The time of obtaining the certificate could affect the return-to-work date. In addition, as the analysis was based on PSWCI data obtained through household interviews during a specific period, recall bias is possible [4, 28, 30, 32].

## Conclusions

In conclusion, injured workers with a certification generally had a higher RTW rate. In particular, the RTW rate was higher among female workers and daily workers with a certification than among those without. This implies that having a certification may play a more important role in RTW among female workers and daily workers, who were relatively vulnerable to lower RTW rates. Therefore, strategies are needed to assist injured workers, especially those belonging to vulnerable groups, in obtaining certifications that facilitate their RTW.

## Data Availability

No data available.
